# Long non-coding RNA ARHGAP5-AS1 inhibits migration of breast cancer cell via stabilizing SMAD7 protein

**DOI:** 10.1007/s10549-021-06286-5

**Published:** 2021-08-09

**Authors:** Chen-Long Wang, Jing-Chi Li, Ci-Xiang Zhou, Cheng-Ning Ma, Di-Fei Wang, Lu-Lu Wo, Ming He, Qianqian Yin, Jian-Rong He, Qian Zhao

**Affiliations:** 1 Department of Pathophysiology, Key Laboratory of Cell Differentiation and Apoptosis of National Ministry of Education, Shanghai Jiao Tong University School of Medicine (SJTU-SM), No. 280, Chong Qing South Rd, Shanghai, 200025 China; 2 Department of Pathology, Xuzhou Medical University, Xuzhou, 221004 China; 3 Comprehensive Breast Health Center, Shanghai Ruijin Hospital, Shanghai Jiao Tong University School of Medicine (SJTU-SM), No. 197, Rui Jin Er Road, Shanghai, 200025 China; 4 Shanghai Institute for Advanced Immunochemical Studies, ShanghaiTech University, Shanghai, 201210 China

**Keywords:** lncRNA, ARHGAP5-AS1, Breast cancer, Metastasis, SMAD7

## Abstract

**Purpose:**

Tumor metastasis is the main cause of death from breast cancer patients and cell migration plays a critical role in cancer metastasis. Recent studies have shown long non-coding RNAs (lncRNAs) play an essential role in the initiation and progression of cancer. In the present study, the role of an LncRNA, Rho GTPase Activating Protein 5- Antisense 1 (ARHGAP5-AS1) in breast cancer was investigated.

**Methods:**

RNA sequencing was performed to find out dysregulated LncRNAs in MDA-MB-231-LM2 cells. Transwell migration assays and F-actin staining were utilized to estimate cell migration ability. RNA pulldown assays and RNA immunoprecipitation were used to prove the interaction between ARHGAP5-AS1 and SMAD7. Western blot and immunofluorescence imaging were used to examine the protein levels. Dual luciferase reporter assays were performed to evaluate the activation of TGF-β signaling.

**Results:**

We analyzed the RNA-seq data of MDA-MB-231 and its highly metastatic derivative MDA-MB-231-LM2 cell lines (referred to as LM2) and identified a novel lncRNA (NR_027263) named as ARHGAP5-AS1, which expression was significantly downregulated in LM2 cells. Further functional investigation showed ARHGAP5-AS1 could inhibit cell migration via suppression of stress fibers in breast cancer cell lines. Afterwards, SMAD7 was further identified to interact with ARHGAP5-AS1 by its PY motif and thus its ubiquitination and degradation was blocked due to reduced interaction with E3 ligase SMURF1 and SMURF2. Moreover, ARHGAP5-AS1 could inhibit TGF-β signaling pathway due to its inhibitory role on SMAD7.

**Conclusion:**

ARHGAP5-AS1 inhibits breast cancer cell migration via stabilization of SMAD7 protein and could serve as a novel biomarker and a potential target for breast cancer in the future.

**Supplementary Information:**

The online version contains supplementary material available at 10.1007/s10549-021-06286-5.

## Introduction

Breast cancer is one of the most common cancer and accounts for 29% (246,660) of all new cancers among the worldwide women [1]. According to the latest statistics, released by the American Cancer Society, about 276,480 new cases of invasive breast cancer are expected to be diagnosed in the USA in 2020, which means one in eight women will get breast cancer during their lifetime. In addition, 42,170 deaths per year makes breast cancer the second leading cause of cancer-related mortality among women in the USA [2]. The main cause of death from breast cancer is due to metastasis to other organs (e.g. bone, lung, brain and liver) [3]. Nevertheless, the mechanisms underlying the metastatic dissemination remain poorly understood, which causes a critical barrier for breast cancer therapy. Cell migration is regarded to be one of the essential step involved in metastasis [4]. Reduced migration suppressed the dissemination of cancer cells in a breast cancer mouse model [5]. It is well characterized that cancer cell could achieve migration via rearrangement of cytoskeleton [6]. Thus, expanding the understanding of mechanisms underlying cytoskeleton reorganization may promote the understanding of cell migration and hence cancer metastasis.

Recent studies have shown that long non-coding RNAs (lncRNAs) play an essential role in the initiation and progression of cancer. LncRNAs are gene transcripts which have more than 200 nucleotides in length and have no potential of translation [7]. LncRNAs have been proposed to carry out diverse functions, including transcriptional regulation *in cis* or *trans*, organization of nuclear domains, and regulation of proteins or RNA molecules [8]. It is now widely understood that lncRNAs could identify cellular pathologies such as cancer, provide prognostic value, or even serve as therapeutic options for cancer patients [9]. For example, over-expression of the lncRNA HOTAIR in early stage, surgically resected breast cancer is highly predictive of progression to metastatic disease and overall survival [10]. Silencing the expression of the lncRNA GUARDIN triggered apoptosis and senescence, enhanced cytotoxicity of additional genotoxic stress and inhibited cancer xenograft growth. Thus, GUARDIN may constitute a target for cancer treatment [11].

The transforming growth factor β (TGFβ) signaling pathway is a key player in metazoan biology, and its misregulation can result in tumor development. Pathological forms of TGFβ signaling promote tumor growth and invasion, evasion of immune surveillance, as well as cancer cell dissemination and metastasis [12]. Smad6 and Smad7 are inhibitory Smads that negatively control TGFβ pathway activity in response to feedback loops and antagonistic signals [13]. SMAD7, as one of the key inhibitors of TGFβ signaling, negatively regulates the whole pathway via multiple mechanisms [14]. In the cytoplasm, SMAD7 can compete the binding site of type-1 TGFβ receptor (TGFβ R1) with SMAD2/3 and hence inhibits signal transduction by blocking phosphorylation of SMAD2/3 [15]. Besides, SMURF1 and SMURF2 are recruited to TGFβ R1 by SMAD7 and induce the degradation of receptors [16]. Meanwhile in the nucleus, SMAD7 disrupts the binding of SMAD2/3/4 complex with DNA [17].

In our present study, we identified a novel lncRNA ARHGAP5-AS1, which expression is downregulated in highly metastatic breast cancer cell line MDA-MB-231-LM2. Functional study showed that ARHGAP5-AS1 could inhibit migration of breast cancer cells through inhibition of stress fibers. Moreover, we found that ARHGAP5-AS1 interacted with SMAD7 and stabilized SMAD7 protein via blocking the interaction between SMAD7 and its E3 ligase (SMURF1 & SMURF2), and hence inhibited its ubiquitination and degradation. Furthermore, ARHGAP5-AS1 could inhibit TGFβ signaling pathway by upregulation of TGFβ R1. Taken together, these findings demonstrate that ARHGAP5-AS1 could serve as a novel biomarker for breast cancer metastasis and a potential target for the treatment in the future.

## Methods

### Cell lines and cell culture

MDA-MB-231 was kindly provided by Ming-Yao Liu (East China Normal University, Shanghai, China). SKBR3 and BT549 were purchased from the cell bank of the Chinese Academy of Science (Shanghai, China). MDA-MB-231-LM2 was cultured in DMEM medium (Hyclone) supplemented with 10% FBS (Gbico). MDA-MB-231 was cultured in Leibovitz L-15 medium (Gibco) supplemented with 10% FBS. SKBR3 and BT549 were cultured in RPMI 1640 (Hyclone) medium supplemented with 10% FBS. All cell lines were incubated at 37 ℃ in a humidified atmosphere of 5% CO_2_ and 95% air except for MDA-MB-231 which were raised in a humidified atmosphere containing 100% air.

### RNA extraction and quantitative real-time PCR

Total RNA of cancer cell line were extracted from Trizol reagents (Invitrogen#15596018) according to the manufacturer’s instructions. cDNA was obtained by AMV reverse transcription system (TAKARA#2621) followed the manufacturer’s protocol. Quantitative real-time PCR was performed with SYBR Green PCR master mix reagent (ABI#4472908) and specific primers for SMAD7-F/R (ATGCTGTGCCTTCCTCCGCTG/CCACGCACCAGTGTGACCGA) and ARHGAP5-AS1-F/R (GGCCCCTGATTCAGTACGTT/GCGTGAACAGGGGTCTTTTG). Data analysis of ARHGAP5-AS1 expression in breast cancer cell lines was normalized by internal control 28S RNA and evaluated using 2^∆∆C^^t^ method, while data analysis of breast cancer tissues was normalized by 18S RNA and presented by − ∆Ct.

### LncRNA in vitro Translation

This experiment was performed according to the manufacturer’s instructions (Promega#L1170). Briefly, 1 μg of circular plasmid pcDNA3.1(+)-ARHGAP5-AS1 and pcDNA3.1(+)-ARHGAP5-AS1-AS were used in the translation reaction. The tubes were incubated 1 h at 30 ℃. Then, 2 μL of reaction liquid was added into 15 μL SDS Loading Buffer and boiled 3 times at 105 ℃ on incubator. Streptavidin-HRP (CST#3999) was utilized to detect products of translation.

### Immunofluorescence staining

F-actin staining was performed according to the manufacturer’s instructions (Invitrogen#R415). Briefly, 1.5 × 10^5^ cells transfected with si-NC/si-ARHGAP5-AS1 or pcDNA3.1(+)-VEC/pcDNA3.1( +)-ARHGAP50AS1 for 48 h were seeded onto coverslips in 24-well culture plate. A total volume of 200 μL containing 4 μL Rhodamine Phalloidin was used per well and incubated 30 min at room temperature. As for SMAD7 staining, the FITC-conjugated antibody (Santa Cruz#sc-365846 FITC) was used at the dilution rate of 1:50 in 1% BSA and incubated overnight. Immunofluorescence pictures were taken by Nikon A1R confocal microscope (Nikon, Kanagawa, Japan).

### Transwell migration assay

Cell migration ability was determined by transwell chambers (Corning#3422). The experimental procedure was described elsewhere [18]. Briefly, 2 × 10^4^ cells transfected with si-NC/si-ARHGAP5-AS1 or pcDNA3.1(+)-VEC/pcDNA3.1(+)-ARHGAP5-AS1 for 48 h were seeded into transwell chambers. 18 h later, migrated cells were staining by crystal violet. Pictures were taken by Nikon Eclipse Ti microscope (Nikon, Kanagawa, Japan).

### *RNA Fluorescent *in situ* hybridization*

This experiment was performed with Fluorescent in Situ Hybridization Kit (Ribo#R11060.1) according to the manufacturer’s protocol. Briefly, 1.5 × 10^5^ MDA-MB-231 cells or LM2 cells was seeded onto cover slides in 24-well culture plate. LncRNA ARHGAP5-AS1 was detected by specific probe with Cy3 labeling. Fluorescence pictures were taken by Nikon Eclipse Ti microscope (Nikon, Kanagawa, Japan).

### Protein microarray screening

Sense and antisense lncRNA were transcribed in vitro with Cy5 labeling. Synthesized lncRNA were incubated with HuProt™ microarray (CDI Laboratories, Inc.) which contained more than 20,000 full-length human proteins with GST tag. This procedure was completed by Wayen biotechnologies (Shanghai), Inc. according to the manufacturer’s instructions. In brief, proteome microarrays were blocked with blocking buffer for 1 h at room temperature before incubation of transcribed lncRNA in blocking buffer with microarray at room temperature for 1 h. TBST followed by MilliQ water were used to wash the microarrays three times for 5 min. Dried slides were scanned using GenePix 4000B. Images were analyzed using GenePix Pro 6.0.

### RNA pull down assays

In vitro transcription of antisense or sense ARHGAP5-AS1 was achieved by T7 RNA Polymerase (Roche#10881767001) according to the manufacturer’s instructions. Meanwhile, 2 μL of Biotin RNA Labeling Mix (Roche#11685597910) was added into the transcription reaction. Cell lysates were prepared by sonication in BufferA [150 mM KCl, 25 mM Tris pH7.4, 5 mM EDTA, 0.5 mM DTT, 0.5% NP40 supplemented with 1 mM PMSF (Bytotime#ST506) and Cocktail (Millipore#539134)]. 15 μg transcribed RNA was added into lysates of 1 × 10^7^ cells and incubated at 4 ℃ for 2 h in a rotary shaker. Streptavidin Magnetic Beads (NEB#S1420) was utilized to enrich RNA–protein complex before washing five times in BufferA and elution in SDS Loading buffer. Eluted proteins were separated by SDS-PAGE for western blot.

### RNA Immunoprecipitation (RIP) assays

RIP assays were performed by utilizing the Millipore Magna RIP Kit (#17-700) according to the manufacturer’s protocol. Bound RNA was extracted by Trizol reagents and subjected to real-time PCR using specific primers.

### Dual luciferase reporter assays

4 × SBE was synthesized and cloned into pGL4.27 vector. 293T cells were cultured in 24-well tissue culture plates for 24 h before transfection. Each three wells were transfected with pGL4.27–4 × SBE/EV, pcDNA3.1(+)-ARHGAP5-AS1/EV and Renilla. 5 ng/mL TGFβ1 was added into each well 48 h after transfection. After 6 h stimulation, 293T cells were lysed by Passive Lysis Buffer. Luciferase activity was detected according to manufacturer’s protocol (Promega#E1910).

### Western blot

This experimental procedure was described elsewhere [18]. Antibodies used in this article: SMAD7 (Sigma-Aldrich#SAB1404041-100UG), β-actin (MBL#PM053-7), LARP1 (ABCAM#AB86359), GAPDH (Santa Cruz#SC-32233), FLAG (Sigma-Aldrich#F1804), SMAD7-B8-FITC (Santa Cruz#SC365846FITC), p-SMAD-2/3 (CST#9510), SAMD2/3 (CST#8685), TGF-βR1(Sigma-Aldrich#SAB4502958-100UG), LaminB (Santa Cruz#SC6216), HIS (Beyotime#AH367).

### Statistical analyses

All experiments were repeated at least three times or otherwise mentioned. The p values for comparison between two groups were obtained by Student’s *t*-test (two-tailed). All values were presented as Mean ± STD and the p value < 0.05 was considered to be statistically significant.

## Results

### ARHGAP5-AS1 is a downregulated LncRNA in aggressive breast cancer cells

**Fig. 1:**
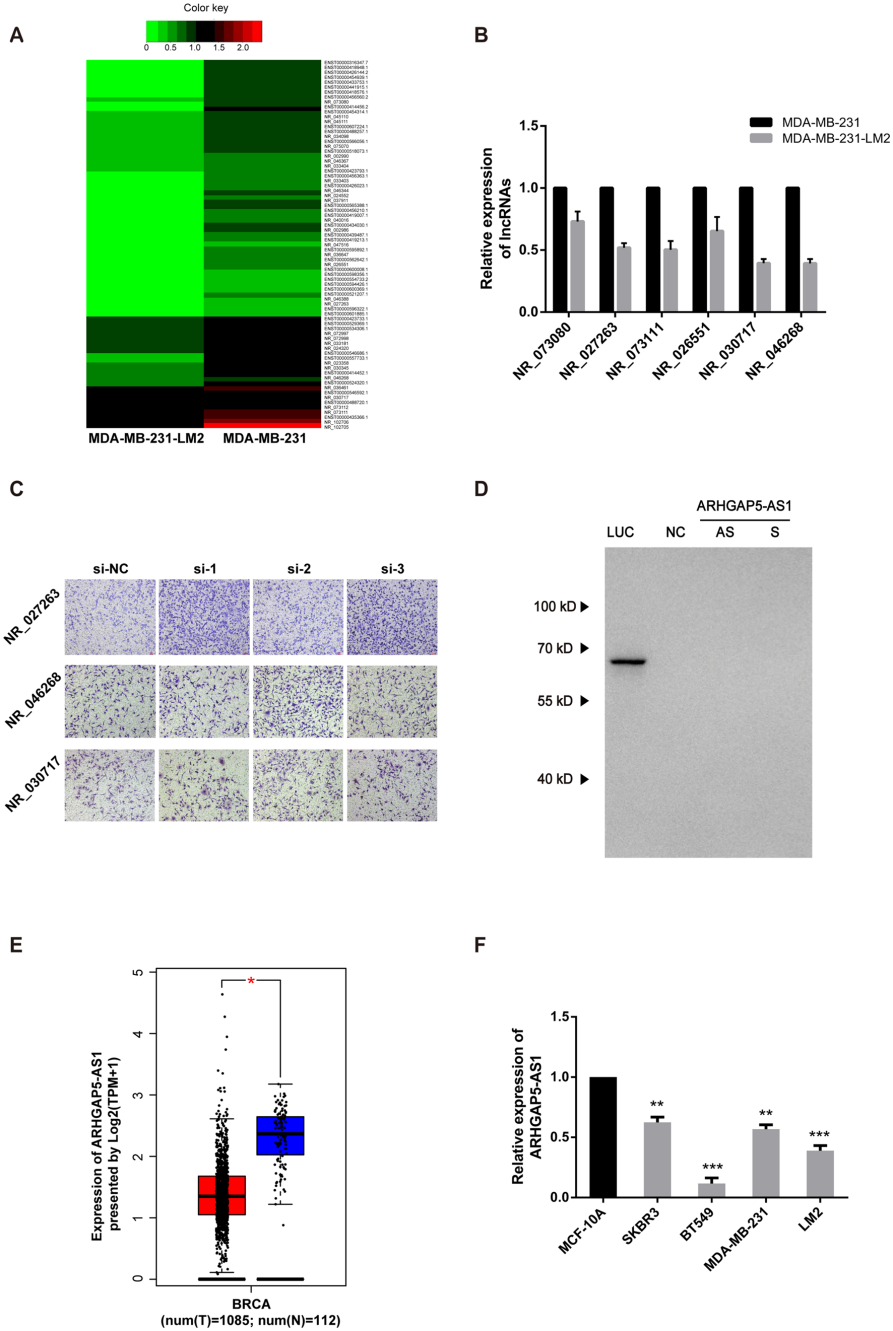
LncRNA ARHGAP5-AS1 is downregulated in aggressive breast cancer tissues and cell lines. **A** Heatmap of dysregulated RNA transcripts in MDA-MB-231 and MDA-MB-231-LM2 were shown. **B** The downregulation level of six LncRNA candidates in MDA-MB-231-LM2 cells was detected by qRT-PCR, compared to MDA-MB-231 cells. **C** The effects of NR_027263, NR_046268 and NR_030717 on the migration ability of MDA-MB-231 cells were detected by transwell migration assays. **D** in vitro translation of plasmids containing luciferase, negative control, antisense and sense ARHGAP5-AS1 were detected by western blot. **E** The differential expression of ARHGAP5-AS1 in BRCA tissues (*n* = 1109) and normal tissues (*n* = 113) from TCGA database was shown. **F** The expression of ARHGAP5-AS1 in human breast cancer cell lines was analyzed by qRT-PCR. The error bars in all graphs represented SD. *p < 0.05, **p < 0.01, ***p < 0.001

To investigate the downregulated LncRNAs involved in breast cancer metastasis, MDA-MB-231-LM2 which is a more aggressive subtype of MDA-MB-231 was utilized [19]. LncRNA expression profiling of MDA-MB-231-LM2 and MDA-MB-231 cells were explored by RNA sequencing (Fig. 1A). To ensure the detectability of lncRNA candidates, we only focused on the transcripts which RPKM is greater than 1. Seventy-nine RNA transcripts were downregulated in MDA-MB-231-LM2 cells (fold change > 2) (Table S1). Among them, 33 RNA transcripts are included in NCBI database, which means the whole length of these transcripts are certain. Since 7 transcripts aren’t non-coding RNA, 11 transcripts cannot be detected by qPCR and 9 transcripts don’t have suitable regions for siRNA design, we successfully validated 6 downregulated lncRNAs in LM2 cells at last (Fig. 1B). Subsequently, 3 lncRNA genes (NR_027263, NR_030717 and NR_046268) was reduced by at least 50% and subjected to functional validation. For each LncRNA candidates, three siRNA fragments were designed and transfected into MDA-MB-231 cells. In order to verify the role of these LncRNAs on metastasis, transwell migration assays were performed. Suppression of NR_027263 showed the highest promotion on cell migration among three candidates (Fig. 1C). According to the genetic location of NR_027263, it was named as Rho GTPase activating protein 5 antisense RNA 1 (ARHGAP5-AS1). To determine the identity of ARHGAP5-AS1, the translational potential of ARHGAP5-AS1 was detected by in vitro translational system and the results showed ARHGAP5-AS1 cannot encode any protein (Fig. 1D), thus it’s a long non-coding RNA. In order to validate the downregulation of ARHGAP5-AS1 in breast cancer specimens, the data were achieved from TCGA database and analyzed by GEPIA (http://gepia.cancer-pku.cn/). The results showed that ARHGAP5-AS1 is downregulated in cancer tissues compared to normal tissues (Fig. 1E). The reduction of ARHGAP5-AS1 was further proved in four breast cancer cell lines, which MCF-10A is utilized as positive control (Fig. 1F). Taken together, the downregulation of ARHGAP5-AS1 promoted cell migration, suggesting a tumor-suppressive role of ARHGAP5-AS1 in breast cancer cells.

### ARHGAP5-AS1 inhibits migration of breast cancer cells

**Fig. 2:**
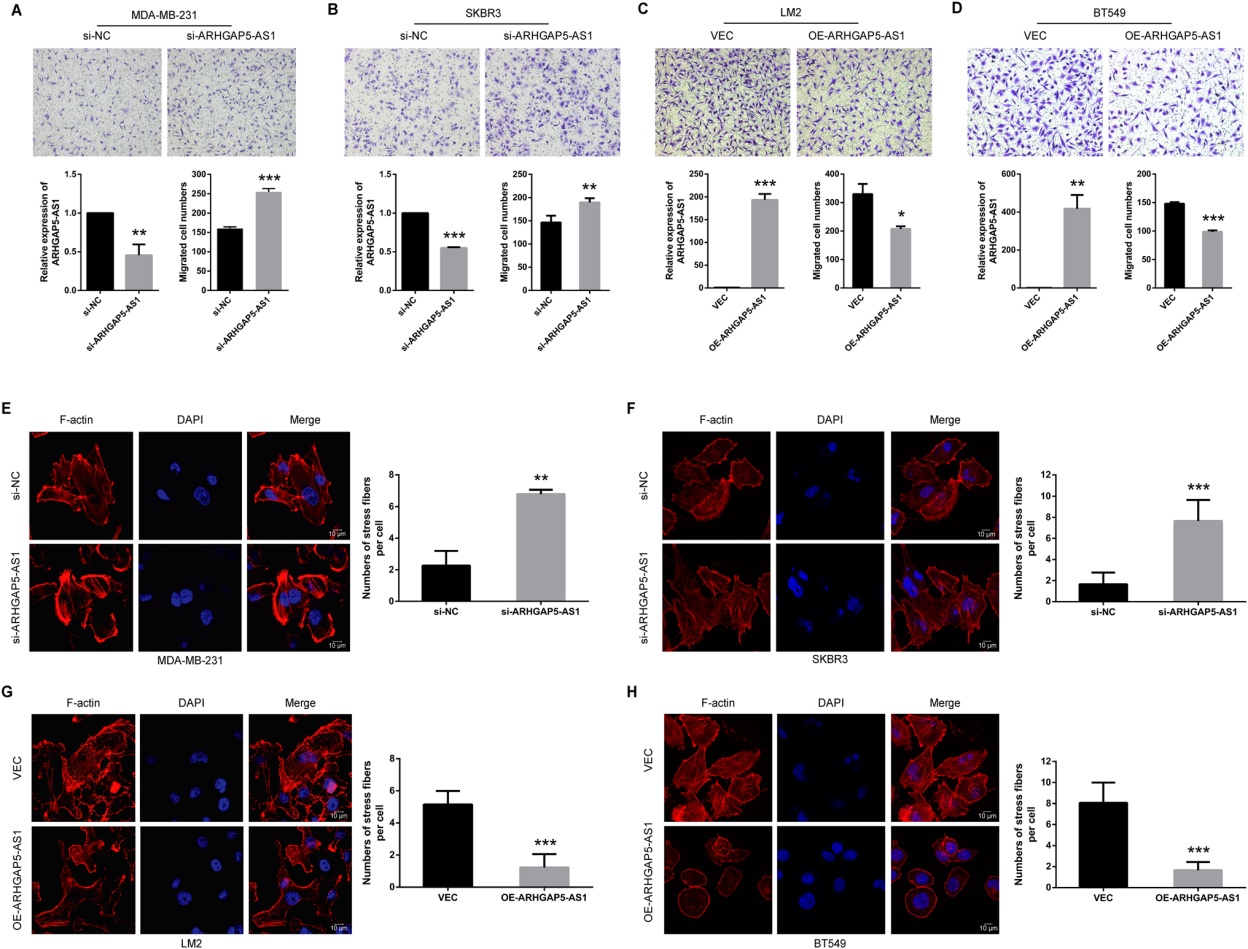
ARHGAP5-AS1 inhibits migration of breast cancer cells. Transwell migration assays of knockdown of ARHGAP5-AS1 in **A** MDA-MB-231 cells and **B** SKBR3 cells as well as over-expression of ARHGAP5-AS1 in **C** LM2 cells and **D** BT549 cells were measured and the results were expressed as the number of migrated cells per field. Knockdown & over-expression efficiencies of ARHGAP5-AS1 in each cell line were shown below. Immunofluorescence of F-actin of knockdown of ARHGAP5-AS1 in **E** MDA-MB-231 cells and **D** SKBR3 cells as well as over-expression of ARHGAP5-AS1 in **F** LM2 cells and **G** BT549 cells were performed and the results were presented as the number of stress fibers per cell. The error bars in all graphs represented SD and each experiment was repeated three times independently. *p < 0.05, **p < 0.01, ***p < 0.001

Besides of MDA-MB-231 and LM2 cells, SKBR3 and BT549 were also utilized to further verify the functional role of ARHGAP5-AS1 in breast cancer. The data showed that knockdown of ARHGAP5-AS1 promoted migration of MDA-MB-231 cells (Fig. 2A) and SKBR3 cells (Fig. 2B). On the contrary, over-expression of ARHGAP5-AS1 inhibited migration of LM2 cells (Fig. 2C) and BT549 cells (Fig. 2D). Interestingly, enlarged lamellopodia was observed in migrated cells of ARHGAP5-AS1 knockdown group of MDA-MB-231 cells, suggesting the reorganization of cytoskeleton. Thus, the distribution of the main component of cytoskeleton—F-actin was detected by immunofluorescence. The results showed that knockdown of ARHGAP5-AS1 expression increased the formation of stress fibers in MDA-MB-231 cells (Fig. 2E) as well as SKBR3 cells (Fig. 2F). Conversely, over-expression of ARHGAP5-AS1 reduce the formation of stress fibers in LM2 cells (Fig. 2G) as well as BT549 cells (Fig. 2H). Taken together, ARHGAP5-AS1 affected migration of breast cancer cells through the alteration of stress fibers formation.

### ARHGAP5-AS1 interacts with the PY motif of SMAD7

**Fig. 3:**
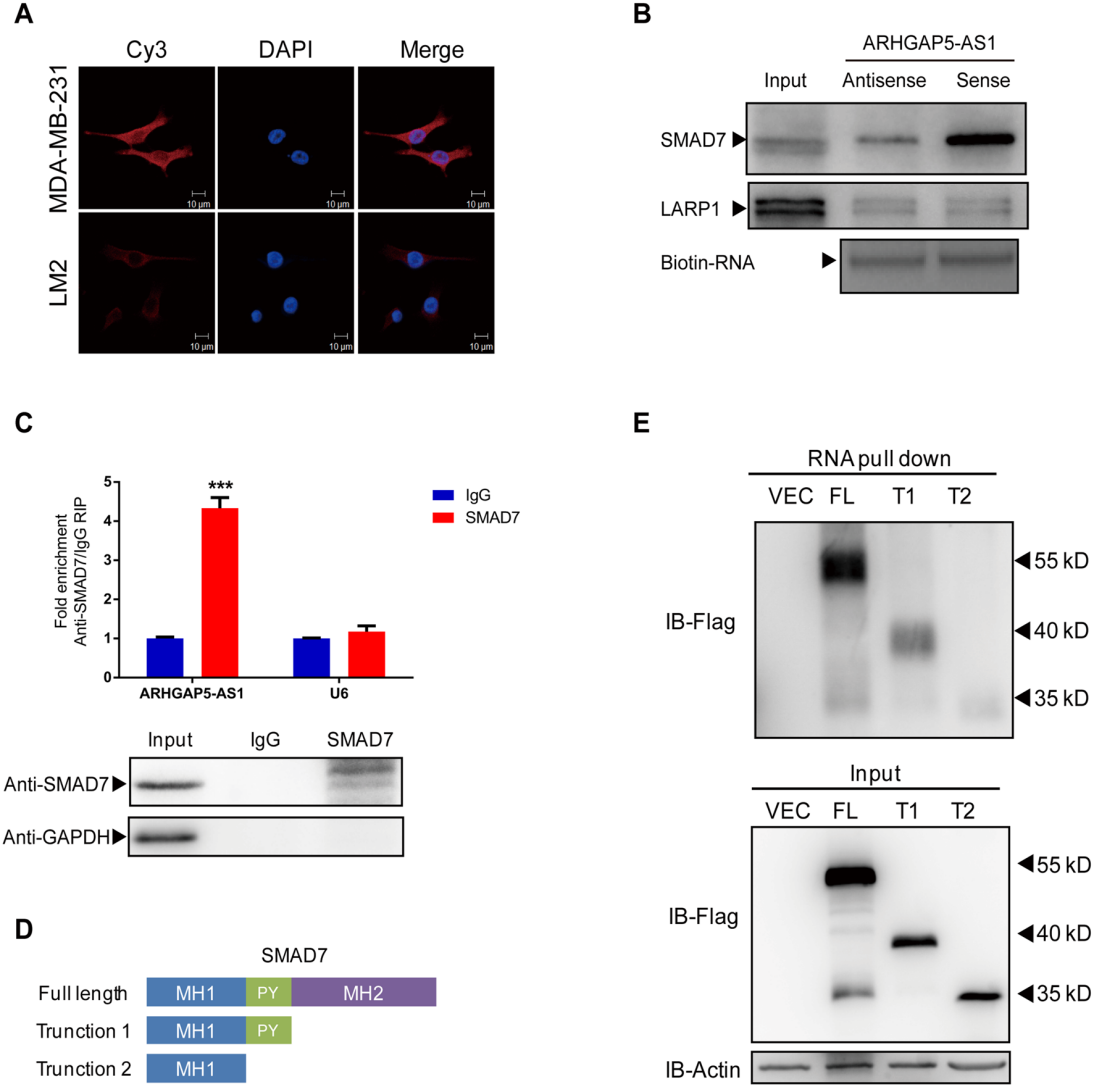
ARHGAP5-AS1 interacts with the PY motif of SMAD7. **A** The subcellular location of ARHGAP5-AS1 in MDA-MB-231 (upper) and LM2 (lower) cells were detected by RNA FISH with Cy3 labeled probes. **B** Identification of ARHGAP5-AS1 interacting proteins by RNA pull down assay was detected by western blot. **C** RNA immunoprecipitation assay was performed using SMAD7 and IgG antibody in MDA-MB-231 cells. Pulled-down ARHGAP5-AS1 and U6 were detected by qRT-PCR. **D** The graph showed structures of full-length SMAD7 and truncation 1 & 2. **E** RNA pull down assay was performed to detect the interaction between ARHGAP5-AS1 and flag-tagged SMAD7, truncation 1 & 2. Each experiment was repeated three times independently

LncRNAs can function as protein scaffold or microRNA sponge to regulate cellular processes in cytoplasm, while regulating gene transcription *in cis* or *trans* in the nucleus [9]. In order to explore the mechanism of ARHGAP5-AS1, the subcellular location was first performed and the results showed that ARHGAP5-AS1 mainly existed in cytoplasm rather than in the nucleus (Fig. 3A), so we focused on the proteins interacting with ARHGAP5-AS1. Based on this result, protein microarray screening was utilized to identify the potential ARHGAP5-AS1-interacting protein. Firstly, sense and antisense ARHGAP5-AS1 were transcripted in vitro with Cy5 labeling (Figure S1A). The screening showed that there were 26 proteins interacting with ARHGAP5-AS1 sense excluding with antisense (Figure S1B). There were five proteins correlated to breast cancer progression among all ARHGAP5-AS1-interacting proteins, and SMAD7 showed highest fluorescence intensity (Figure S1C). SMAD7 was proved to interact with ARHGAP5-AS1 by RNA pull down assay (Fig. 3B). More importantly, the enrichment of SMAD7 and ARHGAP5-AS1 complex could be detected by RNA immunoprecipitation (Fig. 3C). Furthermore, flag-tagged full-length and truncations of SMAD7 were constructed to determine domains mediating the interaction with ARHGAP5-AS1 (Fig. 3D). In detail, truncation 1 abrogated MH2 domain at C-terminal of SMAD7 and truncation 2 remained MH1 domain only at N-terminal. The RNA pull down results showed that both full-length and truncation 1 of SMAD7 were able to interact with ARHGAP5-AS1, but truncation 2 lost the interaction with ARHGAP5-AS1 (Fig. 3E). This result suggested that PY motif was essential for the interaction between ARHGAP5-AS1 and SMAD7. Taken together, ARHGAP5-AS1 interacted with SMAD7 protein through its PY motif.

### *ARHGAP5-AS1 stabilizes SMAD7 *via* inhibition of SMAD7 ubiquitination*

**Fig. 4:**
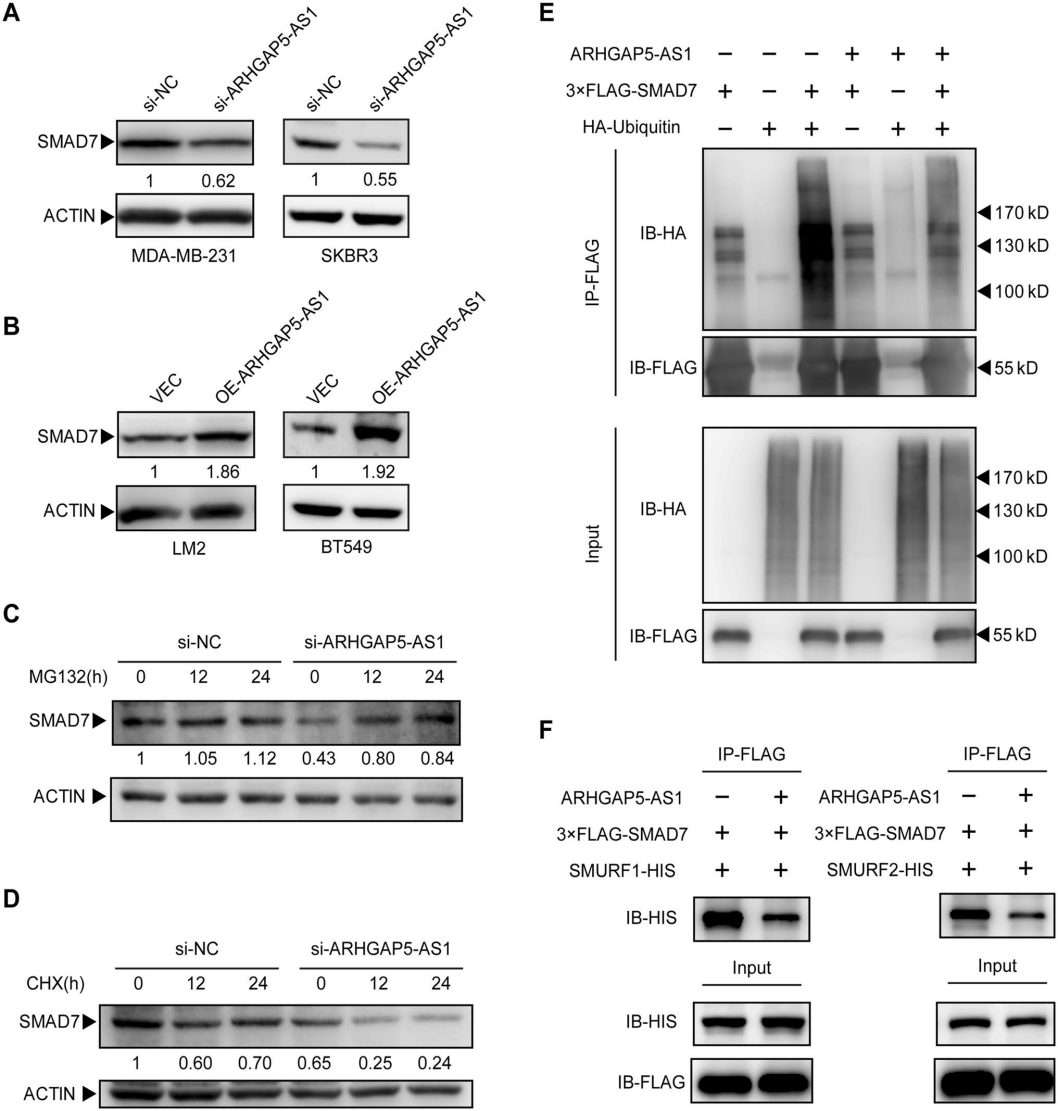
ARHGAP5-AS1 stabilizes SMAD7 via inhibition of SMAD7 ubiquitination. **A** The protein level of SMAD7 was detected by western blot in MDA-MB-231 cells and SKBR3 cells with knockdown of ARHGAP5-AS1. **B** The protein level of SMAD7 was detected by western blot in LM2 cells and BT549 cells with over-expression of ARHGAP5-AS1. **C** Knockdown of ARHGAP5-AS1 in MDA-MB-231 cells with treatment of 20 μM MG132. **D** Knockdown of ARHGAP5-AS1 in MDA-MB-231 cells with treatment of 40 μM CHX. **E** Immunoprecipitation was performed to enrich ubiquitin-SMAD7 complex in 293 T cells with indicated transfections. **F** Immunoprecipitation of FLAG was performed to enrich FLAG-tagged SMAD7 and HIS-tagged SMURF1/SMURF2 complex with or without over-expression of ARHGAP5-AS1 in 293 T cells. Each experiment was repeated three times independently

The PY motif of SMAD7 has been reported to be responsible for the interaction between SMAD7 and its E3 ligase (SMURF1 & 2) and the subsequent degradation of SMAD7 [20]. Hereafter, the effect of ARHGAP5-AS1 on the stability of SMAD7 was investigated. As expected, knockdown of ARHGAP5-AS1 reduced the protein level of SMAD7 in MDA-MB-231 cells and SKBR3 cells, while over-expression of ARHGAP5-AS1 increased the protein level of SMAD7 in LM2 cells and BT549 cells (Fig. 4A and B). The degradation of SMAD7 was controlled by ubiquitin and proteasome [21]. The proteasome inhibitor MG132 was applied to block the effects of ARHGAP5-AS1 on SMAD7. The results showed that MG132 treatment blocked the degradation of SMAD7 induced by knockdown of ARHGAP5-AS1 (Fig. 4C). In order to certify that the reduction of SMAD7 by knockdown of ARHGAP5-AS1 is due to the increase in degradation rather than the decrease in its mRNA level, the relative mRNA level of SMAD7 was detected by qRT-PCR. The results showed that either knockdown or over-expression of ARHGAP5-AS1 had no influence on the mRNA level of SMAD7 (Figure S2A and B). Meanwhile, knockdown of ARHGAP5-AS1 accelerated the degradation of SMAD7 when the protein synthesis inhibitor cycloheximide (CHX) stimulation applied (Fig. 4D). Degradation of proteins in proteasome system results from poly-ubiquitination of proteins, so that the ubiquitination of SMAD7 was detected by immunoprecipitation. The results showed that over-expression of ARHGAP5-AS1 reduced ubiquitination of SMAD7 in 293T cells (Fig. 4E). Moreover, ARHGAP5-AS1 had a negative effect on the interaction between SMAD7 and its E3 ligases SMURF1 and SMURF2 (Fig. 4F). On the contrast, ARHGAP5-AS1 had no effect on the interaction between SMAD7 and its deubiquitinase OTUD1 (Figure S2C). Taken together, ARHGAP5-AS1 stabilized SMAD7 protein through impeding the interaction between SMAD7 and its E3 ligase as well as the subsequent ubiquitination and the degradation of SMAD7.

### SMAD7 mediates the negative effect of ARHGAP5-AS1 on cell migration

**Fig. 5:**
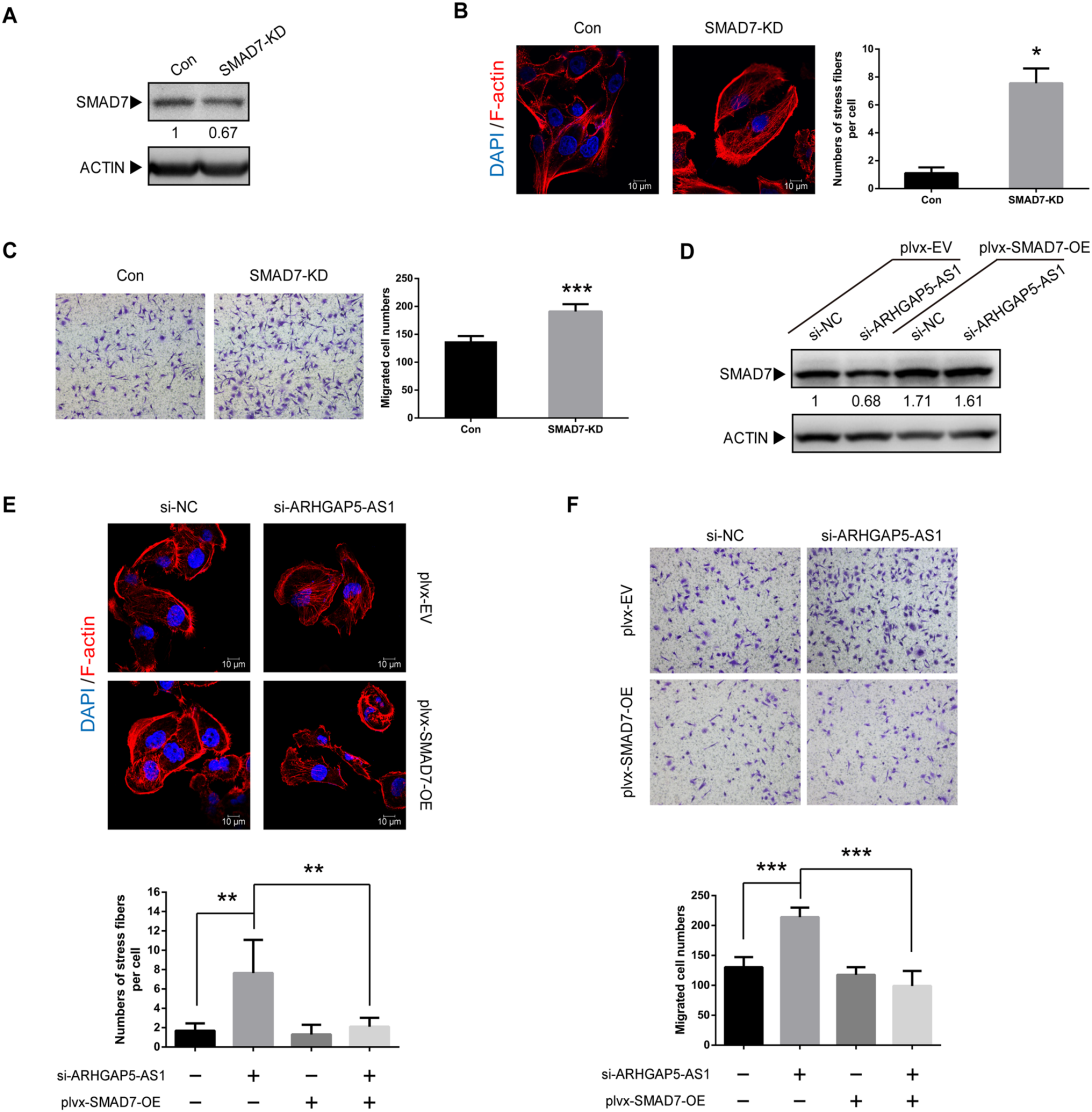
SMAD7 mediates the negative effect of ARHGAP5-AS1 on cell migration. **A** The knockdown efficiency of SMAD7 DNA antisense oligo was detected by western blot. **B** Immunofluorescence of F-actin of SMAD7 knockdown in MDA-MB-231 cells was performed and the results were presented as the number of stress fibers per cell. **C** Transwell migration assay of SMAD7 knockdown was measured and the results were expressed as the number of migrated cells per field. **D** The protein level of SMAD7 was detected by western blot in plvx-EV and plvx-SMAD7-OE cells with si-NC or si-ARHGAP5-AS1 transfection. **E** Immunofluorescence of F-actin was performed with indicated transfections. **F** Transwell migration assay was performed with indicated transfections. The error bars in all graphs represented SD and each experiment was repeated three times independently. *p < 0.05, **p < 0.01, ***p < 0.001

As SMAD7 antagonizes TGFβ signaling, it has critical role in TGF-β induced cytoskeleton rearrangement [22]. In order to determine whether SMAD7 is involved in the suppression of ARHGAP5-AS1 on breast cancer cell migration, antisense DNA oligos was utilized to knockdown SMAD7 which mimicked knockdown of ARHGAP5-AS1. The knockdown efficiency was detected by western blot, and the results showed efficient knockdown of SMAD7 (Fig. 5A). Immunofluorescence by TRITC-phalloidin showed an obvious increase in stress fiber formation (Fig. 5B) and numbers of migrated cells (Fig. 5C) in MDA-MB-231 cells through knockdown of SMAD7, which mimicked the phenotype induced by knockdown of ARHGAP5-AS1. To gain a better understanding of functional role of SMAD7, a lentiviral vector-mediated over-expression of SMAD7 (PLVX- SMAD7-OE) was performed in the ARHGAP5-AS1 knockdown cells (Fig. 5D). The functional assay results showed that the ectopic expression of SMAD7 reversed the promotion of stress fiber formation (Fig. 5E) and cell migration (Fig. 5F) induced by ARHGAP5-AS1 knockdown. In contrast, the over-expression of SMAD7 did not significantly alter either stress fiber formation or cell migration in si-NC transfected cells. Taken together, all the results indicated that SMAD7 mediated the effect of ARHGAP5-AS1 on breast cancer cell migration.

### *Knockdown of ARHGAP5-AS1 promotes TGF-β signaling *via* destabilizing SMAD7*

**Fig. 6:**
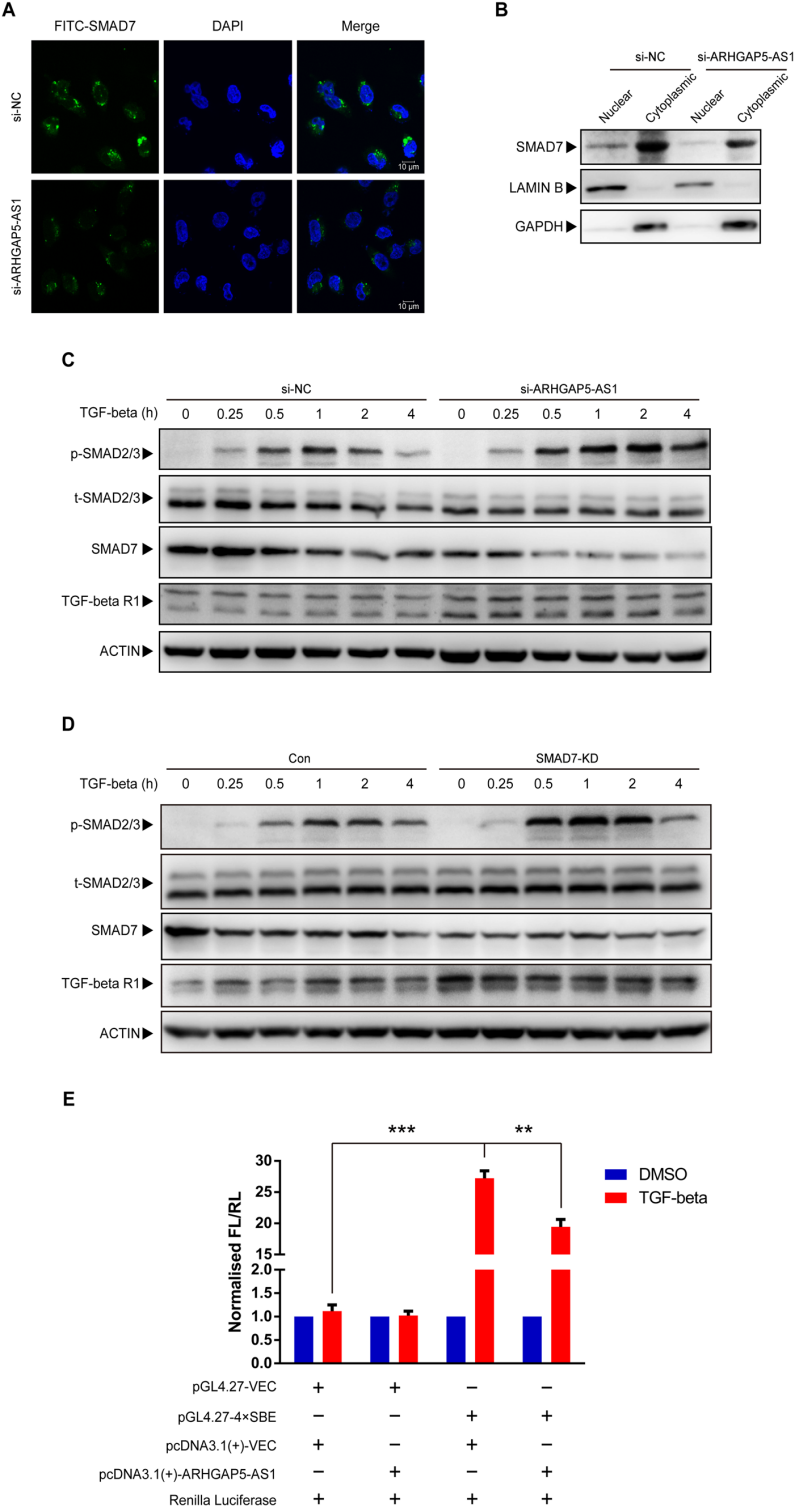
Knockdown of ARHGAP5-AS1 promotes TGF-β signaling via SMAD7. **A** Immunofluorescence of SMAD7 with FITC-conjugated antibody was performed to detect location of SMAD7 in MDA-MB-231 cells. **B** Nuclear and cytoplasmic SMAD7 was detected by western blot, LAMIN B and GAPDH were used as loading controls. **C** Effects of ARHGAP5-AS1 knockdown on the activation of TGF-β signaling were detected by western blot. **D** Effects of SMAD7 knockdown on the activation of TGF-β signaling were detected by western blot. **E** Luciferase reporter assays were performed in 293T cells with indicated transfections. The error bars in all graphs represented SD and each experiment was repeated three times independently. *p < 0.05, **p < 0.01, ***p < 0.001

Due to the different inhibitory mechanisms of SMAD7 in nucleus and cytoplasm [14], the subcellular location of SMAD7 was detected with knockdown of ARHGAP5-AS1. Our data showed that ARHGAP5-AS1 knockdown did not alter the predominant cytoplasmic location of SMAD7, but significantly downregulated SMAD7 protein level in the cytoplasm through immunofluorescence staining (Fig. 6A) as well as western blotting analysis of nuclear and cytoplasmic fractions (Fig. 6B), which was in consistence with the our previous results in Fig. 4A. Based on the evidence that SMAD7 could antagonize TGFβ signaling by inducing the degradation of TGFβ R1 [16], the effects of ARHGAP5-AS1 on TGFβ signaling, the components of TGFβ pathway were investigated. Western blotting analysis showed increased expression of TGFβ R1 and prolonged activation of TGFβ signaling, which in turn presented by increased phosphorylation of SMAD2 in response to TGFβ1 stimulation (Fig. 6C). The similar pattern was showed by knockdown of SMAD7 (Fig. 6D). Moreover, we constructed four SMAD-binding elements (SBE) into pGL4.27 vector and performed dual luciferase reporter assays in 293T cells. The results showed that the activity of firefly luciferase was enhanced by the treatment of TGF-β and over-expression of ARHGAP5-AS1 inhibited the enhancement of luciferase activity (Fig. 6E). Taken together, ARHGAP5-AS1 negatively regulates TGFβ signaling by destabilizing SMAD7 and upregulation of TGFβ R1.

## Discussion

This study provides the first evidence that LncRNA ARHGAP5-AS1 could inhibit cell migration via suppression of stress fibers in breast cancer cells. More importantly, to our knowledge it is the first lncRNA could directly interact with SMAD7, which is an important inhibitory Smads that negatively control TGFβ signaling pathway. The present study demonstrated that ARHGAP5-AS1 impaired the degradation of SMAD7 via interaction with its PY motif, blocking the interaction with its E3 ligase (SMURF1 & SMURF2) and thus decreased its ubiquitination. Knockdown of SMAD7 mimicked the promotion of cell migration by knockdown of ARHGAP5-AS1. Meanwhile, ectopic expression of SMAD7 blocked the increase in migration and stress fiber formation by knockdown of ARHGAP5-AS1. Moreover, ARHGAP5-AS1 inhibited the formation of stress fiber and hence migration via suppression of TGFβ signaling by stabilized SMAD7.

ARHGAP5-AS1 located on the human chromosome 14 and was first identified as a transcriptional isoform of ARHGAP5 in gastric cancer by large-scale sequencing [23]. Non-coding RNA was considered as transcription “noise” in the past. However, more and more studies showed an emerging and diverse role in cancer pathways [9]. For example, lncRNA NKILA suppresses breast cancer metastasis by interaction with p65 and blocking IκB phosphorylation [24]. Here we illustrated that ARHGAP5-AS1 was a long non-coding RNA and its expression was downregulated in aggressive breast cancer cells. Besides, ARHGAP5-AS1 expression was also downregulated in breast cancer tissues compared to normal tissues, suggesting a tumor-suppressor role in breast cancer. Thus, we investigated the function of ARHGAP5-AS1 in progression of breast cancer. As a result, ARHGAP5-AS1 inhibited migration of breast cancer cells. Moreover, stress fiber formation was reduced by ARHGAP5-AS1. Moreover, the correlation between clinical benefit and ARHGAP5-AS1 expression was analyzed and improved recurrence-free survival for Luminal A type patients with a high expression of ARHGAP5-AS1 (Figure S3), which seems to in concordance with the anti-migration effect of ARHGAP5-AS1 in vitro. It’s a pity that we have not studied the regulatory mechanism of ARHGAP5-AS1 expression in breast cancer cells. We thought there could be a possibility that the expression of ARHGAP5-AS1 is regulated by some transcription factors which expression are quite different in breast cancer tissues.

LncRNA functions as protein scaffold, which are already well established [25]. We demonstrated that ARHGAP5-AS1 interacted with PY motif of SMAD7 protein. PY motif is responsible for the interaction of SMAD7 with WW domain of E3 ligase [20]. Indeed, ARHGAP5-AS1 occupied PY motif and blocked the ubiquitination of SMAD7 by E3 ligase (SMRUF1 & SMURF2), so that reduced the degradation of SMAD7 protein. SMAD7 is a powerful inhibitor of TGFβ signaling, antagonizing TGFβ signaling via multiple mechanisms [14]. TGFβ signaling pathway exerts tumor-suppressive effects in cancer initiation, yet it promotes epithelial to mesenchymal transition (EMT), cell invasion as well as migration in cancer progression [26, 27]. The dual role of TGFβ in cancer has long been noted, but its mechanistic basis, operating logic, and clinical relevance have remained elusive. TGFβ induces cytoskeleton reorganization by upregulation of RhoGEFs, such as NET1 [28] and GEFH1 [29]. Besides, SMAD7 inhibits TGFβ induced actin reorganization and RhoA activation [22]. It is expectable that TGFβ induced stress fibers and cell migration could be blocked by ARHGAP5-AS1 due to its interaction with SMAD7 and increased the stability of the latter. However, the function of ARHGAP5-AS1 in breast cancer cells was detected without exogenous TGFβ stimulation. Tumor cells themselves are able to produce TGF-β ligands [30, 31], which might be a reasonable explanation. Indeed, knockdown of SMAD7 mimicked the promotion effect of cell migration by knockdown of ARHGAP5-AS1. Furthermore, ectopic expression of SMAD7 was able to block the promotion of cell migration by knockdown of ARHGAP5-AS1. Above results were also achieved without TGFβ stimulation. To verify the effects of ARHGAP5-AS1 on TGFβ stimulated signaling, we further knockdown the expression of ARHGAP5-AS1 in MDA-MB-231 cells and these cells were starved for 12 h and treated with 0.3 ng/mL TGFβ1. Prolonged activation of TGFβ signaling was demonstrated by upregulation of TGFβ R1 and increased phosphorylation of SMAD2 compared to the control cells. In addition, four fragments of SMAD-binding element (SBE) were cloned into pGL4.27 vector. ARHGAP5-AS1 over-expression showed reduced luciferase activity with stimulation of TGFβ, proving its inhibitory role on TGFβ signaling.

In this study, we give a clear interpretation that ARHGAP5-AS1, which is downregulated in breast cancer tissues and cell lines, suppresses the cell migration and stress fiber formation in breast cancer cells. Furthermore, SMAD7, which is an important inhibitory Smads that negatively control TGFβ signaling pathway, is demonstrated to be interacted with ARHGAP5-AS1 and its protein level is regulated by ARHGAP5-AS1. Thus, our findings provide valuable clues toward understanding the mechanisms of human breast cancer progression and present an opportunity to develop more effective clinical therapies in the future.

## Supplementary Information

Below is the link to the electronic supplementary material.

Supplementary file1 (TIF 2121 kb)

Supplementary file2 (TIF 556 kb)

Supplementary file3 (TIF 577 kb)

Supplementary file4 (DOCX 19 kb)

## Data Availability

The datasets during and/or analyzed the current study available from the corresponding author on reasonable request.

## Abbreviations

LncRNA: Long non-coding RNA
ARHGAP5-AS1: Rho GTPase activating protein 5 antisense RNA 1
TGFβ: Transforming growth factor beta
SBE: SMAD-binding elements
SMURF1 & 2: SMAD specific E3 ubiquitin protein ligase 1 & 2
OTUD1: OTU deubiquitinase 1
