# Supplementary figures and images for: Long non-coding RNA ARHGAP5-AS1 inhibits migration of breast cancer cell via stabilizing SMAD7 protein
Source: Breast Cancer Res Treat. 2021 Aug 9;189(3):607–19. doi: 10.1007/s10549-021-06286-5 (PMC8505316; doi:10.1007/s10549-021-06286-5)

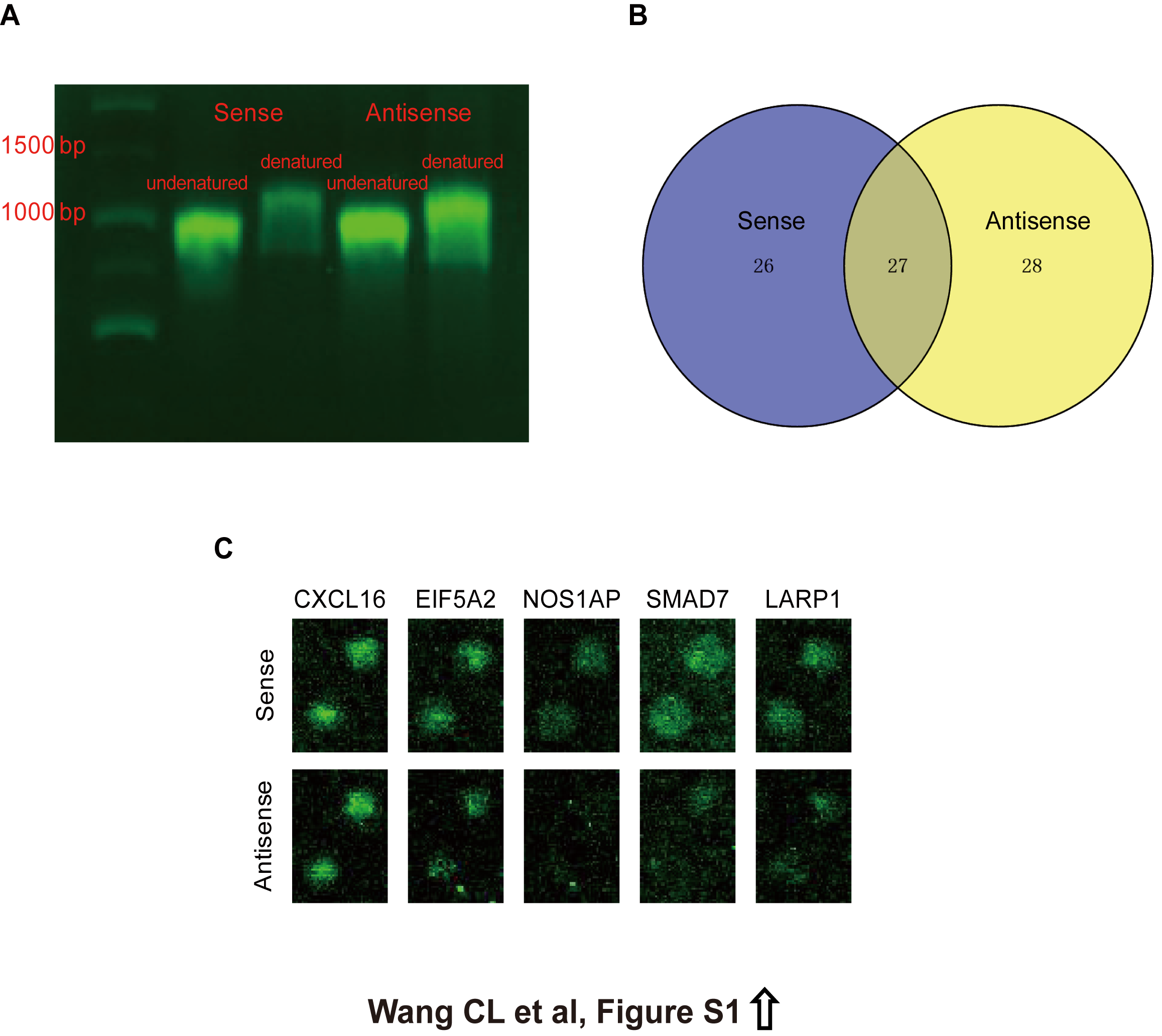

Supplement: Supplementary file 1 — Supplementary file1 (TIF 2121 kb) [file 10549_2021_6286_MOESM1_ESM.tif]

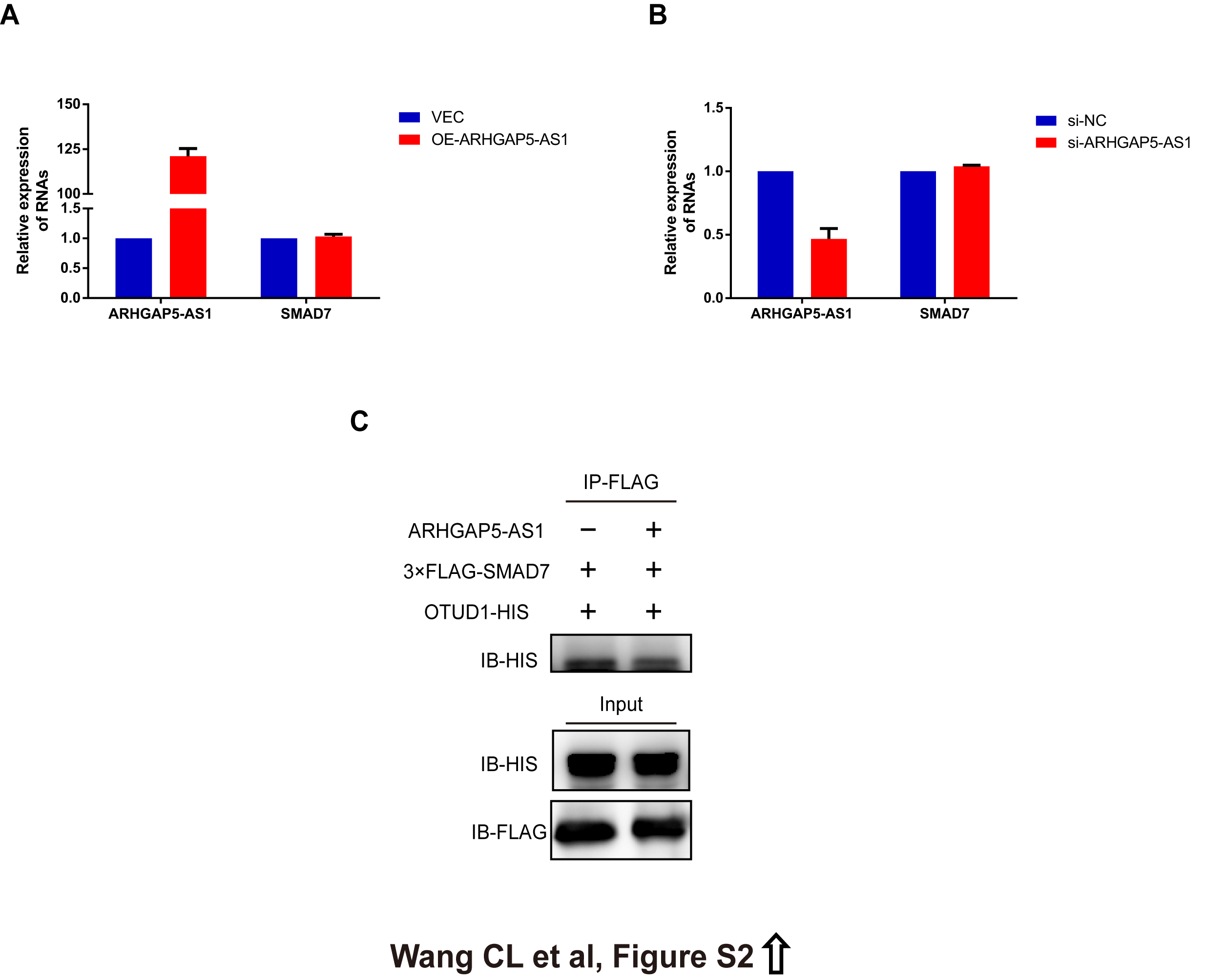

Supplement: Supplementary file 2 — Supplementary file2 (TIF 556 kb) [file 10549_2021_6286_MOESM2_ESM.tif]

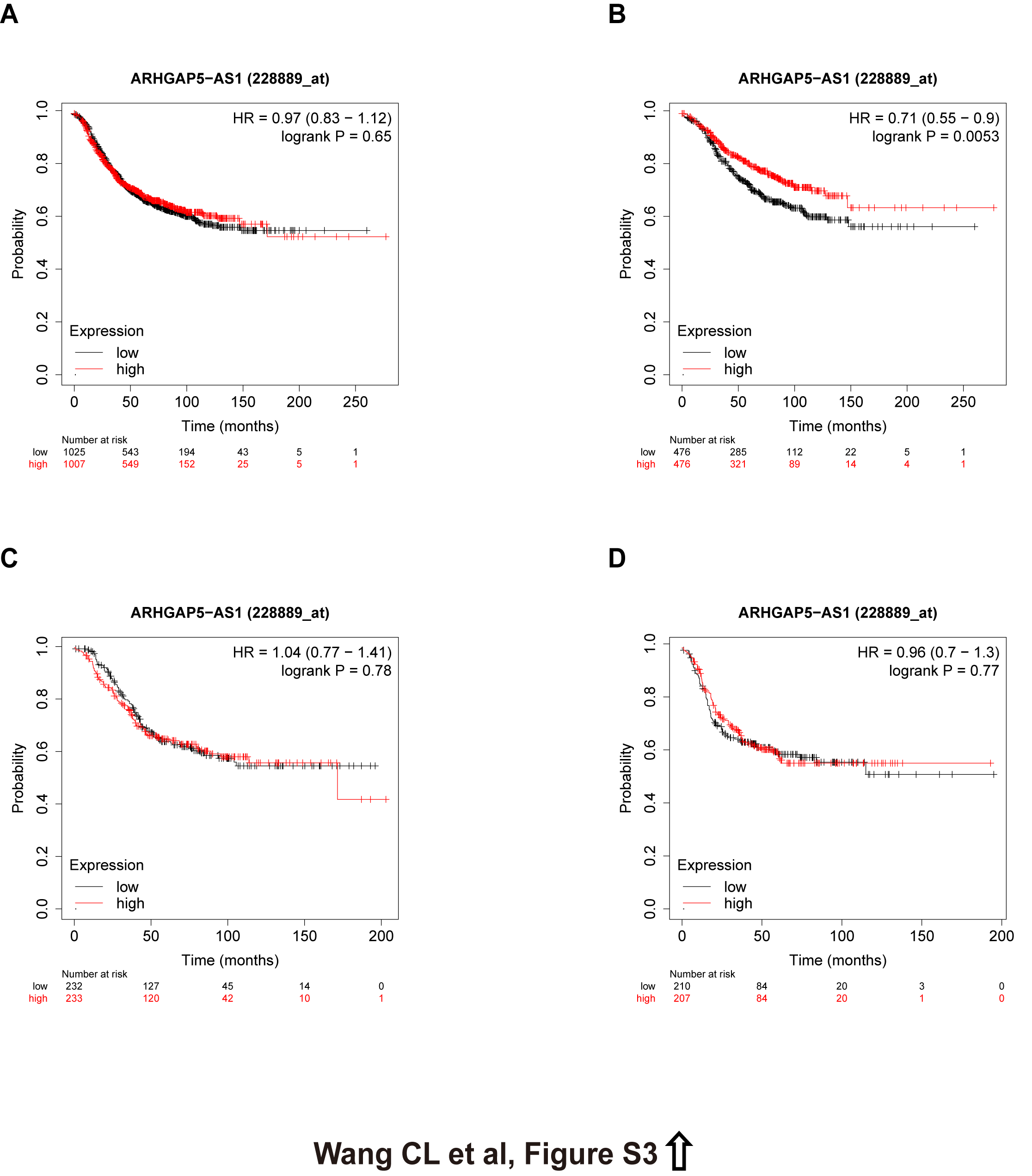

Supplement: Supplementary file 3 — Supplementary file3 (TIF 577 kb) [file 10549_2021_6286_MOESM3_ESM.tif]
