# Supplementary material for: Long non-coding RNA ARHGAP5-AS1 inhibits migration of breast cancer cell via stabilizing SMAD7 protein
Source: Breast Cancer Res Treat. 2021 Aug 9;189(3):607–19. doi: 10.1007/s10549-021-06286-5 (PMC8505316; doi:10.1007/s10549-021-06286-5)
Supplement: Supplementary file 4 — Supplementary file4 (DOCX 19 kb) [file 10549_2021_6286_MOESM4_ESM.docx]

Table S1. RPKM and Fold Change of downregulated lncRNAs in MDA-MB-231-LM2 cells

| lncRNA gene code | Reads Per Kilobase of transcript per Million mapped reads (RPKM) | | Fold Change |
| --- | --- | --- | --- |
|  | MDA-MB-231 | MDA-MB-231-LM2 |  |
| ENST00000316347.7 | 5.38921 | 1.48051 | 3.6401 |
| ENST00000418948.1 | 5.35648 | 1.47085 | 3.6418 |
| ENST00000426144.2 | 5.3806 | 1.41452 | 3.8038 |
| ENST00000454939.1 | 5.1868 | 1.4317 | 3.6228 |
| ENST00000433753.1 | 5.20079 | 1.49691 | 3.4744 |
| ENST00000441915.1 | 5.09928 | 1.47045 | 3.4678 |
| ENST00000418576.1 | 4.97255 | 1.48906 | 3.3394 |
| ENST00000456560.2 | 5.01273 | 1.47306 | 3.4029 |
| NR_073080 | 5.2126 | 1.708 | 3.0519 |
| ENST00000414456.2 | 5.66536 | 1.55623 | 3.6404 |
| ENST00000454314.1 | 6.44623 | 1.48473 | 4.3417 |
| NR_045110 | 4.86929 | 2.28679 | 2.1293 |
| NR_045111 | 4.85975 | 2.28232 | 2.1293 |
| ENST00000607224.1 | 5.32295 | 2.22471 | 2.3926 |
| ENST00000488257.1 | 5.53947 | 2.50867 | 2.2081 |
| NR_034098 | 5.50429 | 2.37218 | 2.3204 |
| ENST00000566056.1 | 4.76637 | 1.97643 | 2.4116 |
| NR_075070 | 4.75746 | 1.88474 | 2.5242 |
| ENST00000518073.1 | 4.40126 | 2.04559 | 2.1516 |
| NR_002990 | 4.34758 | 1.9382 | 2.2431 |
| NR_046367 | 3.5036 | 1.64542 | 2.1293 |
| NR_033404 | 3.47856 | 1.59189 | 2.1852 |
| ENST00000423793.1 | 3.3707 | 1.64529 | 2.0487 |
| ENST00000456363.1 | 3.24118 | 1.6098 | 2.0134 |
| NR_033403 | 3.5753 | 1.48915 | 2.4009 |
| ENST00000426023.1 | 3.46845 | 1.51678 | 2.2867 |
| NR_046344 | 3.22898 | 1.48252 | 2.1780 |
| NR_024552 | 3.21431 | 1.48458 | 2.1651 |
| NR_037911 | 4.09279 | 1.5746 | 2.5993 |
| ENST00000565388.1 | 3.8431 | 1.3969 | 2.7512 |
| ENST00000456210.1 | 4.2242 | 1.284 | 3.2899 |
| ENST00000419007.1 | 4.13469 | 1.22798 | 3.3671 |
| NR_040016 | 3.947 | 1.29668 | 3.0439 |
| ENST00000434030.1 | 3.80695 | 1.15027 | 3.3096 |
| NR_002986 | 3.82587 | 1.14012 | 3.3557 |
| ENST00000439487.1 | 4.89505 | 1.25298 | 3.9067 |
| ENST00000419213.1 | 4.94534 | 1.17164 | 4.2209 |
| NR_047516 | 2.66271 | 1.28529 | 2.0717 |
| ENST00000595892.1 | 2.67279 | 1.23421 | 2.1656 |
| NR_036647 | 2.49925 | 1.22243 | 2.0445 |
| ENST00000562642.1 | 3.08025 | 1.288 | 2.3915 |
| NR_026551 | 2.80205 | 1.34153 | 2.0887 |
| ENST00000600008.1 | 2.79975 | 1.17049 | 2.3919 |
| ENST00000598356.1 | 2.7627 | 1.1008 | 2.5097 |
| ENST00000554733.2 | 3.01321 | 1.03617 | 2.9080 |
| ENST00000594426.1 | 2.32879 | 1.08962 | 2.1372 |
| ENST00000600369.1 | 2.30491 | 1.06885 | 2.1564 |
| ENST00000521207.1 | 2.22953 | 1.04816 | 2.1271 |
| NR_046388 | 2.41817 | 1.0337 | 2.3393 |
| NR_027263 | 2.46695 | 1.01071 | 2.4408 |
| ENST00000596322.1 | 2.51931 | 1.16767 | 2.1576 |
| ENST00000601885.1 | 2.49178 | 1.12456 | 2.2158 |
| ENST00000423733.1 | 2.38157 | 1.15202 | 2.0673 |
| ENST00000529369.1 | 2.35178 | 1.15152 | 2.0423 |
| ENST00000534306.1 | 2.05402 | 1.01482 | 2.0240 |
| NR_072997 | 12.0209 | 5.43094 | 2.2134 |
| NR_072998 | 11.7712 | 5.37322 | 2.1907 |
| NR_033181 | 11.8627 | 5.91853 | 2.0043 |
| NR_024320 | 13.6494 | 5.16547 | 2.6424 |
| ENST00000546686.1 | 13.1476 | 4.42362 | 2.9721 |
| ENST00000557733.1 | 10.6212 | 4.55226 | 2.3332 |
| NR_023358 | 9.89449 | 4.4779 | 2.2096 |
| NR_030345 | 11.1375 | 4.3071 | 2.5858 |
| ENST00000414452.1 | 7.50063 | 2.43579 | 3.0793 |
| NR_046268 | 7.50063 | 2.43579 | 3.0793 |
| ENST00000524320.1 | 6.68737 | 2.62698 | 2.5456 |
| NR_036461 | 9.58439 | 2.63755 | 3.6338 |
| ENST00000546592.1 | 6.62031 | 3.29311 | 2.0104 |
| NR_030717 | 6.02832 | 2.9736 | 2.0273 |
| ENST00000488720.1 | 7.50939 | 3.54091 | 2.1208 |
| NR_073112 | 16.2391 | 6.869 | 2.3641 |
| NR_073111 | 15.6239 | 6.5957 | 2.3688 |
| ENST00000435366.1 | 15.46 | 7.16941 | 2.1564 |
| NR_102706 | 14.6018 | 6.77916 | 2.1539 |
| NR_102705 | 14.5116 | 6.76873 | 2.1439 |
| NR_003003 | 19.1384 | 7.53181 | 2.5410 |
| ENST00000424358.1 | 16.7773 | 8.35191 | 2.0088 |
| ENST00000457332.1 | 25.4109 | 10.5294 | 2.4133 |
| ENST00000560769.1 | 77.5521 | 11.3119 | 6.8558 |
